# Neuromyelitis Optica Spectrum Disorder: A Rare Case of Transverse Myelitis and Autonomic Dysfunction

**DOI:** 10.7759/cureus.38791

**Published:** 2023-05-09

**Authors:** Turki F Bugshan, Muhannad Asiri, Mohammed Alqahtani, Rayan Maghrabi, Hessah S Alotaibi, Naif Alharbi

**Affiliations:** 1 Neurology, King Fahad General Hospital, Jeddah, SAU; 2 Neurology, Armed Forces Hospital - Southern Region, Khamis Mushait, SAU

**Keywords:** neuromyelitis optica spectrum disorder, letm, nmo, nmosd, neuromyelitis optica spectrum disorder (nmosd)

## Abstract

Neuromyelitis optica spectrum disorder (NMOSD) is a demyelinating central nervous system disease commonly presenting with optic neuritis and transverse myelitis. Its pathology is mediated by serum aquaporin 4 immunoglobulin G (AQP4-IgG) and myelin oligodendrocyte glycoprotein (MOG) antibodies. It can present in a relapsing and monophasic pattern and is diagnosed using the diagnostic criteria published in 2015 by the international panel on neuromyelitis optica (NMO) diagnosis. We describe the case of a 25-year-old man who had a history of painful eye movement and complete loss of vision affecting his left eye for which he was diagnosed with optic neuritis two months prior to presentation. The patient presented with transverse myelitis followed by a picture of autonomic dysfunction in the form of labile blood pressure and heart rate readings associated with profuse sweating as well as significant MRI findings. Neuromyelitis optica was diagnosed with positive AQP4-IgG and longitudinally extensive transverse myelitis. Treatment was initiated with pulse steroid and plasmapheresis followed by oral prednisolone and azathioprine following which the patient's condition stabilized.

## Introduction

Neuromyelitis optica spectrum disorder (NMOSD) is a demyelinating central nervous system disease mediated by antibodies [1]. The pathology of neuromyelitis optica (NMO) involves binding of aquaporin 4-IgG (AQP4) to AQP4 water pump channels on astrocyte foot processes, activating complement cascade and causing astrocyte injury followed by secondary damage to oligodendrocytes and neurons [2]. The diagnostic criteria as published in 2015 by the International Panel for NMO Diagnosis (IPND) classified NMO into AQP4-seropositive and AQP4-seronegative disease, In AQP4-antibody-seropositive NMOSD, only one core clinical characteristic (optic neuritis, acute myelitis or brain stem syndrome) is required for diagnosis if alternative diagnoses are excluded. In contrast, for the diagnosis of AQP4-antibody-seronegative NMOSD, at least two clinical core characteristics are required with additional supportive MRI findings [3]. In this case report we present a 25-year-old male who presented with transverse myelitis with autonomic dysfunction and significant MRI findings.

## Case presentation

**Figure 1:**
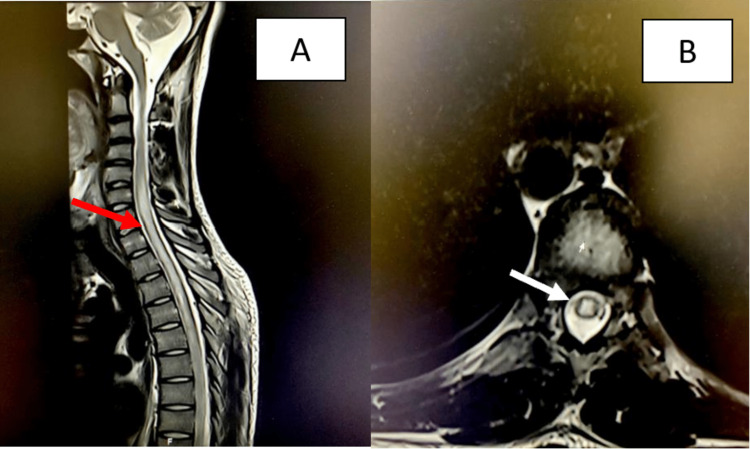
MRI of the spine Cervical and thoracic sagittal (A) and axial (B) T2-weighted MRI shows longitudinal extensive myelitis located centrally within the spinal cord from C5 to T10 intramedullary lesions as indicated by the red and white arrows.

Our patient was a 25-year-old male not known to have any medical illness before developing a history of gradually progressive paraplegia and sensory level reaching T4 dermatome with urine incontinence for 14 days and two months before presentation. The patient experienced painful eye movements and complete loss of vision affecting his left eye for which he was diagnosed with optic neuritis at the time. He was treated with oral steroids and recovered completely within two weeks. There was no history of recent trauma and no family history of autoimmune diseases or similar presentations. Upon examining the patient on admission, he was alert and oriented (AAO)x4 and vitally stable. His upper limbs and lower limbs were significant for spastic hypertonia with power according to the Medical Research Council (MRC) muscle testing scale: 3/5 in the right upper limb, 2/5 in the left upper limb; 3/5 in the right lower limb, 2/5 in left lower limb equal in proximal and distal muscles, and +3 in triceps, biceps, ankle and knee reflexes. In the sensory examination, pinprick sensation was not appreciated below the T4 level, however, the remaining sensations were intact. Coordination and cranial nerve examinations were unremarkable. Complete blood count, blood chemistry (including electrolytes, renal, and liver profiles), and coagulation profile were within normal levels. An MRI brain showed no lesions suggestive of demyelinating disease, while an MRI whole spine showed longitudinal extensive myelitis located centrally within the spinal cord from C5 to T10 intramedullary lesions (Figure 1).

The patient was started on pulse steroids (intravenous methylprednisolone 1g/day) and a lumbar puncture was done for AQP4 IgG, anti-myelin oligodendrocyte glycoprotein (MOG) antibodies, and oligoclonal bands. The samples were sent for cell-based assay as well as serum antibody titers. On day 2 of admission, the patient started sweating profusely and became hypertensive with blood pressure ranging from 105-160/80-110mmhg and tachycardia with a heart rate of 65 to 130. An ECG showed alternating sinus tachycardia and bradycardia, blood gases were within normal. The patient was shifted to the ICU and started plasma exchange with a total of five sessions every other day. During the patient's stay in ICU, his vitals stabilized and his neurological examination was significant for power in the right lower limb 1/5 and left lower limb 0/5. An MRI brain was unremarkable. After finishing the last plasma exchange session, the patient was shifted to the inpatient ward and started on oral prednisolone 60mg once daily and azathioprine 50mg twice daily (which was started due to financial reasons after counseling the patient regarding other treatment options) with physiotherapy sessions. Five days later the patient clinically improved the power in his upper limbs to 4/5 bilaterally, the right lower limb was 3/5, and 2/5 in the left lower limb. His urinary incontinence was completely restored after bladder training and autonomic symptoms improved as well. The patient's CSF results showed cell count, protein, and glucose within normal range. However, AQP4 IgG titer was 1:400, anti-MOG antibodies <1:10, and oligoclonal bands were negative. The patient was discharged home on oral prednisolone and azathioprine after explaining his diagnosis, the benefit and cost of his treatment, and follow-up plans in physiotherapy and outpatient departments.

## Discussion

Neuromyelitis optica is an idiopathic autoimmune demyelinating disease that mainly involves the spinal cord and optic nerves [4,5]. The clinical features of the disorder are severe optic neuritis and longitudinally extensive transverse myelitis which can occur simultaneously or can be separated. Optic neuritis could affect the optic nerve either unilaterally or bilaterally and can happen either before or after a myelitis attack. In 1870, the association of optic neuritis and spinal cord affection was first mentioned by Clifford Albutt, and in 1984 the term 'neuromyelitis optica' was suggested by Eugene Devic and his student Fernand Gault after they presented several cases with a history of simultaneous myelitis attacks and bilateral optic neuritis [6]. In 2006, the international consensus diagnostic criteria for NMOSD were presented as two definitive criteria i.e., transverse myelitis and optic neuritis, and two of the following: brain MRI that is atypical for multiple sclerosis (MS), or AQP4-IgG positivity, or a large spinal cord lesion that exceeds three segments. The term 'NMO spectrum disorders' was proposed for cases that do not fully fulfill these criteria [7]. In the year 2015, these criteria were revised and NMO and the spectrum disorders were accepted as a single entity and named as NMOSD. As per the new criteria, for cases where AQP4- IgG is positive, one main clinical finding is enough for the definitive diagnosis, however, the other possible diagnoses have to be excluded. The main clinical findings are as follows: (1) optic neuritis, (2) acute myelitis, (3) area postrema syndrome, (4) acute brainstem syndrome, (5) symptomatic narcolepsy or acute diencephalic clinical syndrome, (6) symptomatic cerebral syndrome that includes typical brain lesions for NMOSD [8]. These criteria helped clinicians distinguish between NMOSD and other autoimmune diseases such as anti-MOG disease and MS which carried great importance in the management of patients. Brain lesions in NMO cases are usually atypical for MS appearing as ink-blotch lesions, pencil-thin ependymal enhancement, and confluent corpus callosum lesions as well as others, and are usually located in the hypothalamus, brainstem, or cerebral convexity [9].

The auto-antibody specific to the AQP4 water channels associated with the autoimmune inflammatory disease NMO is considered to be an accurate serum biomarker for NMO pathology [10]. This antibody is extremely helpful for confirmation of NMO because of its high specificity (91%; 85% to 99%) and sensitivity (73%; 58 to 76%) [11], and they are also found in patients with longitudinally extensive transverse myelitis without optic neuritis, which is thought to be a precursor to NMO in some cases [12]. A longitudinally extensive cord lesion is considered one of the major supportive diagnostic criteria for NMO, patients with myelitis should have a spinal cord MRI scan with intravenous gadolinium administration to determine whether there is a longitudinally extensive cord lesion. The radiological feature of an NMO cord lesion is usually a contiguous spinal cord lesion 3 or more segments in length with centrally predominant distribution [13]. 

In our patient, an MRI of the whole spine was done and showed longitudinal extensive myelitis located centrally within the spinal cord from C5 to T10 intramedullary lesion, which is an unusual length of longitudinal transverse myelitis (LETM). This MRI showed 13 consecutive vertebral segments as one contiguous spinal cord lesion. Cardiovascular autonomic dysfunction is a common secondary complication of high-thoracic spinal cord disease. Cord lesions involving the T6 level or above, develop autonomic dysreflexia in the form of fluctuating blood pressure and orthostatic hypotension. The frequency of autonomic dysreflexia varies from 7.5% in the acute stage to 90% in the chronic stage following traumatic, severe, cervical/high-thoracic spinal cord injury [14]. Case reports of cardiovascular autonomic dysfunction after primary or metastatic neoplasm and demyelinating disease involving the spinal cord have been documented in the literature [15,16]. In our case, because of LETM which involved the T6 segment and above, the patient had autonomic dysfunction manifestations in the form of profuse sweating, labile high blood pressure readings, and fluctuating heart rate without corresponding factors or related medications, which was managed conservatively.

Neuromyelitis optica is a disease with an aggressive course and needs to be treated aggressively in both acute and maintenance therapies. In an acute attack, pulse steroid therapy is considered a first-line choice. If the patient does not respond to steroid therapy, plasmapheresis, and intravenous immune globulin (IVIG) as well as cytotoxic drugs may be used [17]. In addition, rituximab is considered one of the choices to treat NMO; rituximab is a monoclonal antibody against B lymphocytes [18]. In our case, clinical improvement was observed after the administration of pulse steroids (1g/day of methylprednisolone for five days) followed by five sessions of plasma exchange as management in the acute period. Thereafter the patient was discharged from the hospital with 1mg/kg/day of prednisolone and azathioprine 2 mg/kg/day as maintenance treatment and regular rituximab therapy (every six months). To manage this case, we followed the primary and secondary treatment strategies for NMOSDs which consisted of azathioprine and rituximab combined with oral prednisolone as first-line treatments, while mycophenolate mofetil, mitoxantrone, and cyclophosphamide were considered secondary-line treatment taking into consideration the availability of these drugs and financial barriers [6]. 

## Conclusions

In conclusion, extensive LETM could present in NMO cases with 13 consecutive vertebral segments as one contiguous spinal cord lesion, or a subsequent complication of LETM involving T6 and above as autonomic dysfunction. Prompt diagnosis and early initiation of aggressive immunosuppressive treatment are advised in NMO cases.
